# Accurate prediction of microvascular invasion occurrence and effective prognostic estimation for patients with hepatocellular carcinoma after radical surgical treatment

**DOI:** 10.1186/s12957-022-02792-y

**Published:** 2022-09-30

**Authors:** Yuling Xiong, Peng Cao, Xiaohua Lei, Weiping Tang, Chengming Ding, Shuo Qi, Guodong Chen

**Affiliations:** 1 Hengyang Medical School, University of South China, Hengyang, 421001 Hunan China; 2 Department of Hepatopancreatobiliary Surgery, The First Affiliated Hospital, Hengyang Medical School, University of South China, Hengyang, 421001 Hunan China

**Keywords:** Microvascular invasion, Hepatocellular carcinoma, Survival analysis, Prediction model, Therapeutic strategy

## Abstract

**Background:**

Hepatocellular carcinoma (HCC) is the third most common cause of cancer death worldwide, with an overall 5-year survival rate of less than 18%, which may be related to tumor microvascular invasion (MVI). This study aimed to compare the clinical prognosis of HCC patients with or without MVI after radical surgical treatment, and further analyze the preoperative risk factors related to MVI to promote the development of a new treatment strategy for HCC.

**Methods:**

According to the postoperative pathological diagnosis of MVI, 160 study patients undergoing radical hepatectomy were divided into an MVI-negative group (*n* = 68) and an MVI-positive group (*n* = 92). The clinical outcomes and prognosis were compared between the two groups, and then the parameters were analyzed by multivariate logistic regression to construct an MVI prediction model. Then, the practicability and validity of the model were evaluated, and the clinical prognosis of different MVI risk groups was subsequently compared.

**Result:**

There were no significant differences between the MVI-negative and MVI-positive groups in clinical baseline, hematological, or imaging data. Additionally, the clinical outcome comparison between the two groups presented no significant differences except for the pathological grading (*P* = 0.002) and survival and recurrence rates after surgery (*P* < 0.001). The MVI prediction model, based on preoperative AFP, tumor diameter, and TNM stage, presented superior predictive efficacy (AUC = 0.7997) and good practicability (high H-L goodness of fit, *P* = 0.231). Compared with the MVI high-risk group, the patients in the MVI low-risk group had a higher survival rate (*P* = 0.002) and a lower recurrence rate (*P* = 0.004).

**Conclusion:**

MVI is an independent risk factor for a poor prognosis after radical resection of HCC. The MVI prediction model, consisting of AFP, tumor diameter, and TNM stage, exhibits superior predictive efficacy and strong clinical practicability for MVI prediction and prognostication, which provides a new therapeutic strategy for the standardized treatment of HCC patients.

## Introduction

Hepatocellular carcinoma (HCC) is the sixth most common malignant tumor and the third most common cause of cancer death worldwide [1, 2]. At present, comprehensive treatment based on radical surgery is considered the most effective treatment for HCC [3]. However, even when radical excision is achieved, metastasis and recurrence frequently occur, affecting approximately 70% of patients within 5 years after resection, resulting in a 5-year survival rate of less than 18% [4, 5]. Therefore, an accurate prediction tool is urgently needed to predict the outcome of HCC in the preoperative period, and guide appropriate treatment to improve patient outcomes.

A risk prediction model based on clinical characteristics could play an important role in predicting the outcome of HCC and providing significant guidance for the comprehensive treatment of HCC patients [6, 7]. Several risk factors, including vascular invasion, maximum tumor diameter (MTD), and tumor differentiation, are associated with a high recurrence rate in HCC patients after radical resection [8, 9]. Vascular invasion includes both macrovascular invasion and microvascular invasion (MVI), which is also known as microvascular tumor thrombus, and mainly refers to the nests of tumor cells in the vascular lumen lined by endothelial cells that are visible under the microscope, with and the presence of more than 50 suspended tumor cells per nest [10, 11]. Studies have confirmed that MVI is a predictor of a poor outcome after surgical resection or liver transplantation and is a clinical turning point affecting the postoperative survival of HCC patients [12, 13]. However, the gold standard for clinical MVI detection is still the histopathological examination of postoperative specimens, resulting in the loss of the best treatment opportunity for most HCC patients via a pre-surgical definite diagnosis of MVI. Hence, strengthening the screening and evaluation of independent risk factors for MVI can allow early monitoring of the recurrence and metastasis of tumor cells and create an opportunity for timely retreatment of HCC patients.

**Fig. 1:**
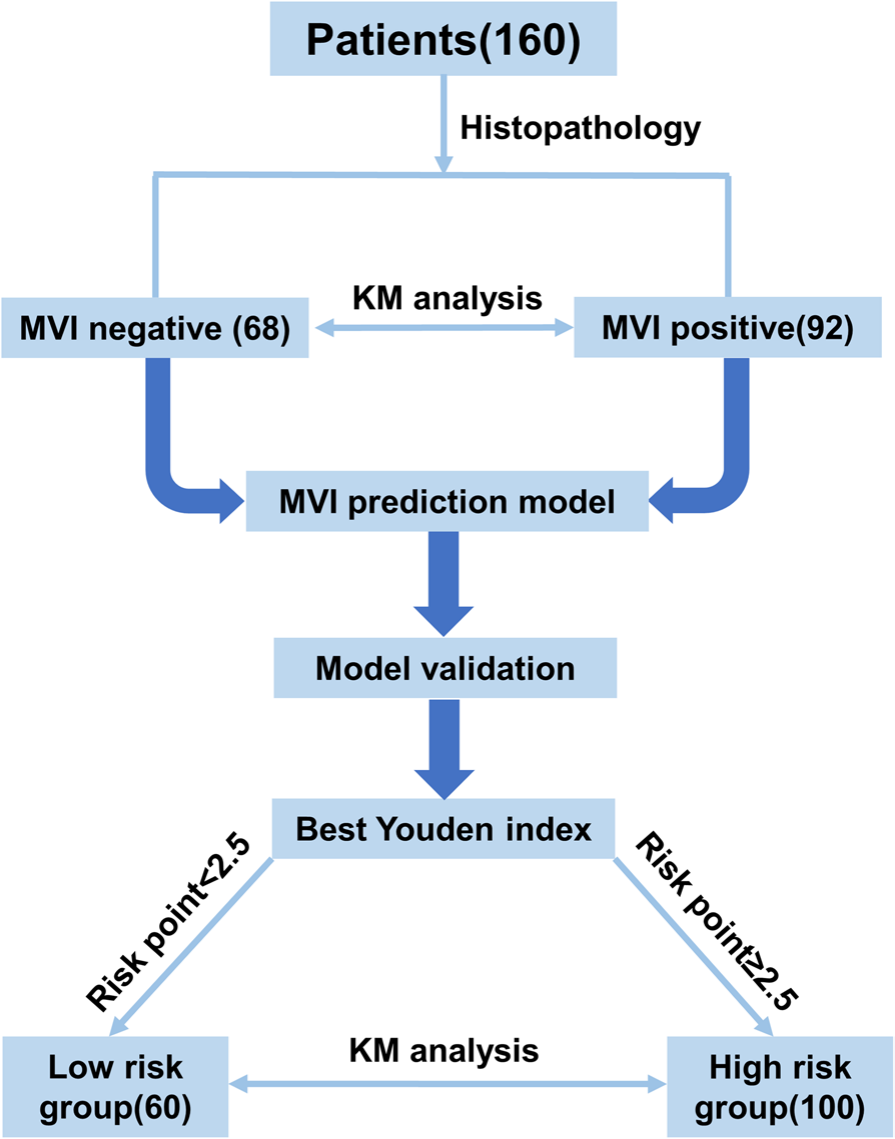
The specific research process of the study

In this study, a retrospective study was conducted to analyze the difference between survival and recurrence in HCC patients with or without MVI undergoing radical surgical treatment, and an MVI prediction model was successfully constructed and validated by analyzing the clinical risk factors associated with MVI. Subsequently, to further verify the validity of this model, all patients were divided into high-risk and low-risk groups for survival analysis. The specific research process is presented in Fig. 1. The model demonstrated great clinical significance for preoperative MVI risk assessment and prognostication of HCC, and it will play an important role in guiding treatment strategies and improving the outcomes of HCC patients.

## Patients and methods

### Study population

A total of 160 patients admitted to the Department of Hepatopancreatobiliary Surgery in the First Affiliated Hospital of University of South China from January 2016 to December 2018 for surgical treatment of liver cancer were enrolled as the study subjects. The patients in this study all underwent anatomic and radical resection by a technical surgical group with more than 500 cases of anatomic and radical hepatectomy experience. The inclusion criteria for all patients were as follows: (1) the preoperative imaging examination was consistent with the imaging characteristics of malignant liver tumors; (2) the preoperative Child-Pugh grading standard of liver function was grade A or B; (3) postoperative pathological diagnosis of HCC; and (4) complete clinical data. The exclusion criteria were (1) previous malignant tumor and surgical history; (2) no obvious surgical indications for advanced malignant tumors: (3) pregnancy; (4) complicated with other serious infectious diseases; and (5) postoperative pathological diagnosis of the primary lesion as a benign liver lesion. This study was approved by the Ethics Committee of the First Affiliated Hospital of University of South China, and all patients who participated in the study signed the corresponding informed consent form.

### MVI diagnostic criteria, grouping, and clinical outcome comparison

The MVI assessment of all study patients was based on postoperative pathology, which was diagnosed by two experienced pathologists. MVI was defined as [14]: nests of cancer cells can be observed under a light microscope that are distributed in the vascular lumen lined by endothelial cells, mainly in the intracapsular vessels of the tumor or the portal vein branches in the liver tissue adjacent to or surrounding the cancer. All study patients were classified into the MVI-positive group and the MVI-negative group. The clinical outcomes between the two groups were compared, including intraoperative data (operation time, bleeding, blood transfusion, and conversion to laparotomy) and postoperative data (hematological indices, incidence of complications, hospital stays, pathological grading, and overall survival/recurrence). The postoperative hematological indices mainly included blood biochemistry tests on postoperative days 1, 3, and 5 (POD1, POD3, and POD5).

### Analysis of the clinical characteristics

The clinical characteristics mainly included the basic clinical data, hematological data and imaging data. The basic clinical data were age, sex, HBV infection, liver cirrhosis, diabetes, hypertension, and smoking; the preoperative hematological data were carcinoma embryonic antigen (CEA), alpha-fetoprotein (AFP), alanine transaminase (ALT), aspartate aminotransferase (AST), total bilirubin (TBIL), albumin (ALB), and prothrombin time (PT); the preoperative imaging data were tumor diameter, number of tumors, and TNM stage (tumor stage confirmed by imaging examination). Based on the previous literature [8, 15], the classification criteria for each candidate index were determined: age (< 60 years or ≥ 60 years), CEA (< 5 ng/mL or ≥ 5 ng/mL), AFP (< 400 ng/mL or ≥ 400 ng/mL), ALT (< 40 U/L or ≥ 40 U/L), AST (< 34 U/L or ≥ 34 U/L), TBIL (< 34 mmol/L or ≥ 34 mmol/L), ALB (< 33 g/L or ≥ 33 g/L), PT (< 13 s or ≥ 13 s), tumor diameter (< 5 cm or ≥ 5 cm), number of tumors (< 2 or ≥ 3), and TNM stage (I–II, III–IV).

### Follow-up evaluation

Follow-up was carried out mainly by telephone, supplemented by systematic inquiry of the patients’ health records. The end of follow-up was defined as 36 months after discharge from admission for HCC resection. Follow-up was conducted until December 2021. The patients were followed up every 3 months during the first postoperative year, and every 6 months for the next 2 years. Follow-up mainly include survival, tumor recurrence, whether there were any relevant treatment, and so on. The median follow-up duration was 22.8 months (range 2 to 36 months).

### Statistical analysis

SPSS 25.0 software was used for statistical analysis. Measurement data are presented as the mean ± standard deviation, and an independent sample *t* test was performed for measurement data subject to a normal distribution. The chi-square test and Fisher’s exact test were used to analyze the statistical data. Univariate analysis and multivariate analysis with the logistic regression proportional hazard model were performed to evaluate the MVI risk factors. The Hosmer-Lemeshow (H-L) test was used to evaluate the goodness of fit of the MVI prediction model. Overall survival and overall recurrence were defined as the time from primary resection until death or censoring, which were calculated by the Kaplan-Meier analysis, and the difference between groups was assessed by the log-rank test. All *P* values were two-sided, with statistical significance set at *P* values less than 0.05.

## Results

### Patient characteristics

All study patients were classified into an MVI-negative group (*n* = 68) and an MVI-positive group (*n* = 92), and the preoperative baseline data comparison between the two groups is shown in Table 1. There were no significant differences between the two groups in clinical baseline data including age, sex, HBV infection, liver cirrhosis, diabetes, hypertension, and smoking (*P* > 0.05). The preoperative hematological data, including CEA, ALT, AST, TBIL, ALB, and PT, also presented no significant differences (*P* > 0.05), while AFP showed a significant difference (222.4 ± 678.7 ng/mL vs. 1227.6 ± 3112.0 ng/mL, *P* = 0.039). There were no significant differences between the two groups in preoperative imaging data including tumor diameter, number of tumors, and TNM stage (*P* > 0.05).

**Table 1:** Preoperative baseline data comparison between the two groups

Variable	MVI negative (***n*** = 68)	MVI positive (***n*** = 92)	***P*** value
**Age (years)**	57.5 ± 9.4	54.3 ± 12.8	0.194
**Gender**			
Female	62 (91.2)	72 (78.3)	0.140
Male	6 (8.8)	20 (21.7)	
**HBV infection**			
Present	18 (26.5)	20 (21.7)	0.159
Absent	50 (73.5)	72 (78.3)	
**Liver cirrhosis**			
Present	32 (47.1)	36 (39.1)	0.492
Absent	36 (52.9)	56 (60.9)	
**Diabetes**			
Present	15 (22.1)	23 (25)	0.605
Absent	53 (77.9)	69 (75)	
**Hypertension**			
Present	12 (17.6)	23 (25)	0.288
Absent	56 (82.4)	69 (75)	
**Smoking**			
Present	13 (19.1)	20 (21.7)	0.753
Absent	55 (80.9)	72 (78.3)	
**CEA (ng/mL)**	3.15 ± 0.89	3.22 ± 1.06	0.659
**AFP (ng/mL)**	222.4 ± 678.7	1227.6 ± 3112.0	**0.039**
**ALT (U/L)**	40.9 ± 32.1	54.5 ± 48.4	0.160
**AST (U/L)**	49.3 ± 39.2	54.3 ± 45.8	0.685
**TBIL (mmol/L)**	15.9 ± 7.1	16.7 ± 8.8	0.664
**ALB (g/L)**	37.4 ± 3.6	38.1 ± 4.6	0.433
**PT (s)**	13.5 ± 1.1	13.7 ± 1.0	0.232
**Tumor diameter (cm)**	4.6 ± 3.2	5.8 ± 3.3	0.095
**Number of tumors**	1.2 ± 0.3	1.6 ± 0.4	0.751
**TNM stage**			
I–II	28 (41.2)	25 (27.8)	0.063
III–IV	40 (58.9)	67 (72.2)	

### The clinical outcome comparison between the two groups

The comparative analysis of the clinical data between the two groups is shown in Table 2. There were no significant differences between the two groups in intraoperative data, including operation time, intraoperative bleeding, intraoperative blood transfusion and the incidence of conversion to laparotomy (*P* > 0.05). The incidence of postoperative complications between the two groups, including surgical site infection (SSI), lung infection, pleural effusion, biliary fistula, and seroperitoneum presented no significant differences (*P* > 0.05), and no patient in the two groups experienced postoperative abdominal bleeding or hepatic failure. No significant difference in the postoperative hospital stay was found between the two groups (*P* > 0.05). However, a difference in pathological grading was observed between the two groups: 32 (47.1%) cases of poor differentiation were found in the MVI-negative group, and 76 (82.6%) cases were found in the MVI-positive group, with a significant difference (*P* = 0.002).

**Table 2:** Comparative analysis of clinical data between the two groups

Variable	MVI negative (***n*** = 68)	MVI positive (***n*** = 92)	***P*** value
**Operation time (min)**	127.9 ± 52.5	149.6 ± 46.0	0.054
**Intraoperative bleeding (mL)**	164.1 ± 155.9	190.9 ± 160.8	0.305
**Intraoperative blood transfusion**	4 (5.9)	14 (15.2)	0.288
**Conversion to laparotomy**	5 (7.4)	12 (13.0)	0.306
**Postoperative complications**			
Surgical site infection	0 (0.0)	2 (2.17)	0.387
Lung infection	12 (17.7)	14 (15.2)	0.770
Pleural effusion	16 (23.5)	20 (21.7)	0.850
Biliary fistula	5 (7.4)	12 (13.0)	0.306
Seroperitoneum	4 (5.9)	8 (8.7)	0.560
Abdominal bleeding	0 (0.0)	0 (0.0)	N/A
Hepatic failure	0 (0.0)	0 (0.0)	N/A
**Postoperative hospital stays (day)**	11.2 ± 3.5	12.0 ± 4.7	0.439
**Pathological grading**			
High/moderate	36 (52.9)	16 (17.4)	**0.002**
Poor	32 (47.1)	76 (82.6)	

**Fig. 2:**
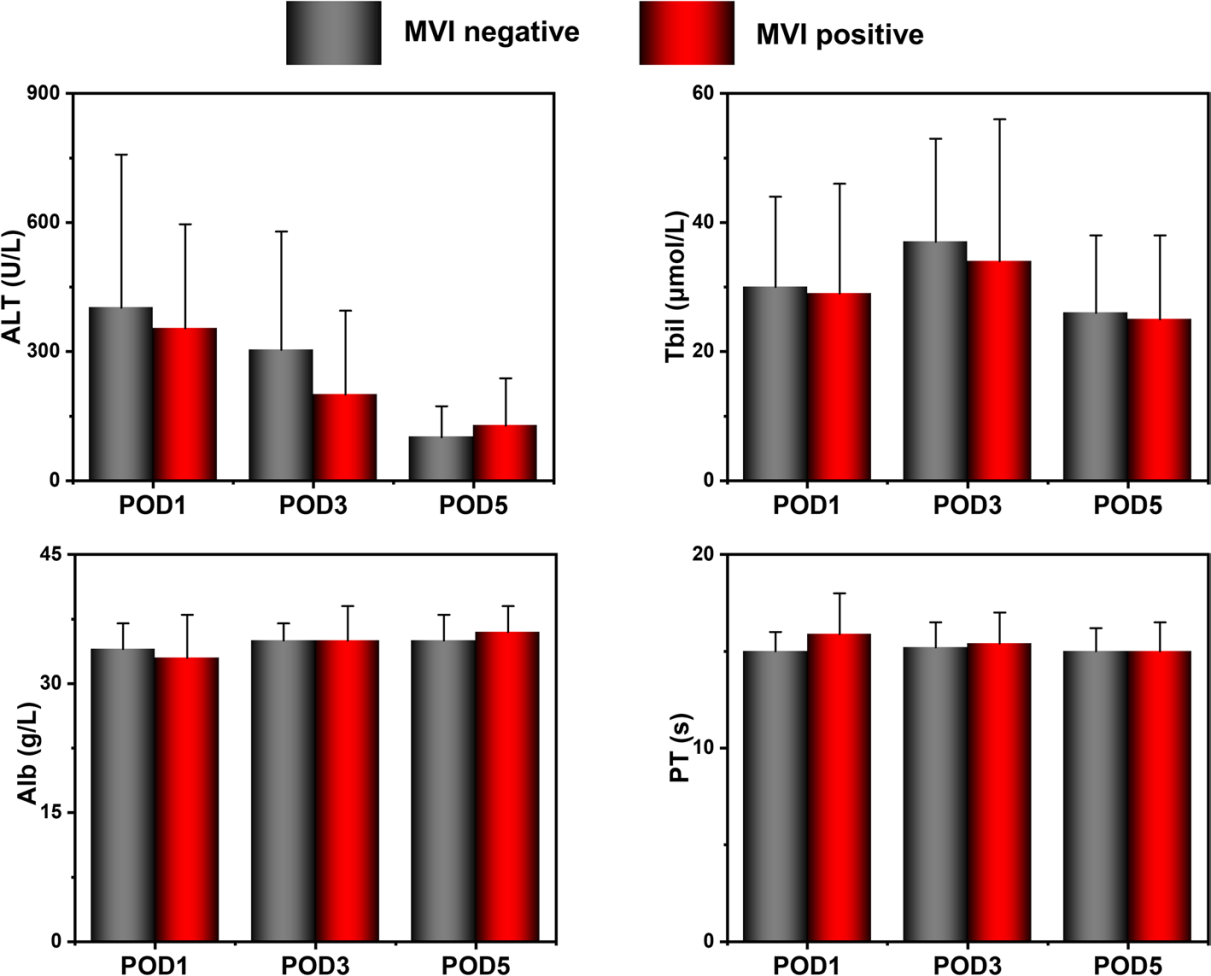
The blood biochemistry comparison between the MVI negative group and MVI positive group 1, 3, and 5 days after surgery

**Fig. 3:**
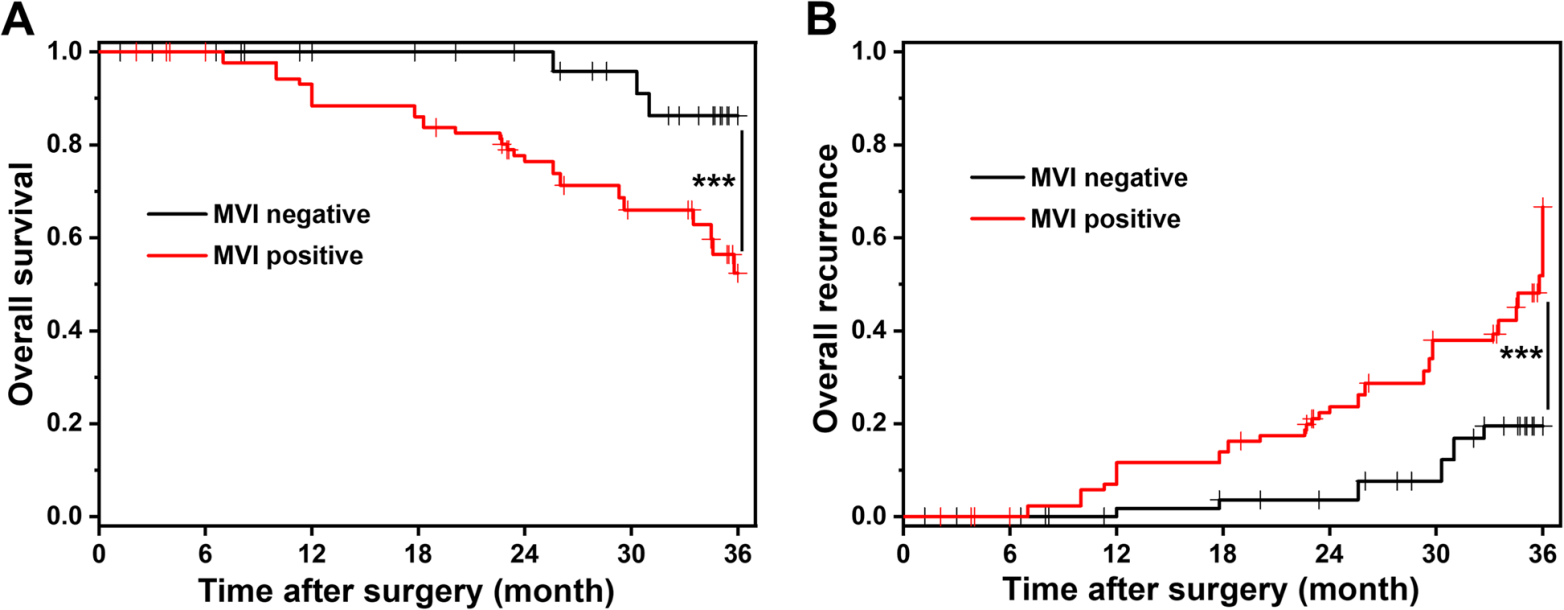
The overall survival (**A**) and recurrence (**B**) comparison between MVI negative group and MVI positive group 36 months after surgery. ^***^P < 0.001

The blood biochemistry comparison between the two groups 1, 3, and 5 days after the operation is shown in Fig. 2. There were no significant differences between the two groups in the postoperative hematological indices including ALT, TBIL, ALB, and PT (*P* > 0.05). The overall survival and recurrence comparison between the two groups 36 months after surgery is presented in Fig. 3. Compared with the MVI-positive group, the patients in the MVI-negative group presented a better survival rate and lower recurrence rate (*P* < 0.001), indicating better clinical outcomes.

### Construction of the MVI prediction model

The clinical characteristics, including the basic clinical data, hematological data, and imaging data were screened by univariate and multivariate logistic regression analyses, which are shown in Table 3. These risk factors, including AFP ≥ 400 kU/L (OR 0.276, *P* = 0.003), TBIL ≥ 34 mmol/L (OR 0.422, *P* = 0.015), tumor diameter (TD) ≥ 5 cm (OR 0.423, *P* = 0.022), and TNM stage (OR 0.238, *P* < 0.001), were screened by univariate logistic regression analysis. After multivariate logistic regression analysis, the significant risk factors, including AFP ≥ 400 kU/L (OR 0.072, *P* < 0.001), tumor diameter (TD) ≥ 5 cm (OR 0.400, *P* = 0.041), and TNM stage III–IV (OR 0.094, *P* < 0.001), were selected to construct a logistic regression model (Table 4). After adding the total number of points scored for each of the three risk factors, the MVI prediction model was 4–3 × AFP – 1 × TD – 2 × TNM. To distinguish the MVI low-risk groups and high-risk groups from all study patients, according to a best Youden index of 0.624, we obtained an optimal cutoff value of 2.5.

**Table 3:** Univariable and multivariate Logistic regression analyses of risk factors for presence of MVI

Variable	Univariate analysis			Multivariate analysis		
	OR	95%CI	***P*** value	OR	95%CI	***P*** value
**Age ≥ 60**	1.516	0.802–2.868	0.200			
**Gender**	0.675	0.357–1.278	0.228			
**HBV infection**	1.447	0.755–2.773	0.266			
**Liver cirrhosis**	1.383	0.733–2.607	0.317			
**Diabetes**	1.134	0.571–2.252	0.719			
**Hypertension**	1.209	0.645–2.264	0.554			
**Smoking**	0.879	0.449–1.722	0.707			
**CEA ≥ 5 ng/mL**	0.614	0.326–1.157	0.131			
**AFP ≥ 400 kU/L**	0.276	0.117–0.649	**0.003**	.0.074	0.027–0.207	**< 0.001**
**ALT ≥ 40 U/L**	0.579	0.260–1.288	0.180			
**AST ≥ 34 U/L**	0.574	0.265–1.246	0.160			
**TBIL ≥ 34 mmol/L**	0.422	0.211–0.844	**0.015**	0.481	0.209–1.107	0.085
**ALB ≥ 33 g/L**	1.850	0.930–3.678	0.079			
**PT ≥ 13 s**	0.764	0.406–1.438	0.404			
**Tumor diameter ≥ 5 cm**	0.423	0.202–0.885	**0.022**	0.395	0.163–0.955	**0.039**
**Number of tumors ≥ 3pcs.**	1.021	0.518–2.015	0.952			
**TNM stage (III–IV stage)**	0.238	0.116–0.486	**< 0.001**	0.099	0.042–0.236	**< 0.001**

**Table 4:** Multivariable analysis of risk factors of MVI and measurement of the risk score

Variable	Multivariate analysis			***B*** coefficient	
	OR	95%CI	***P*** value		Points
**AFP ≥ 400 kU/L**	0.072	0.026–0.198	**< 0.001**	2.630	3
**TD ≥ 5 cm**	0.400	0.166–0.963	**0.041**	0.917	1
**TNM (III–IV stage)**	0.094	0.040–0.223	**< 0.001**	2.360	2

### Model validation

**Fig. 4:**
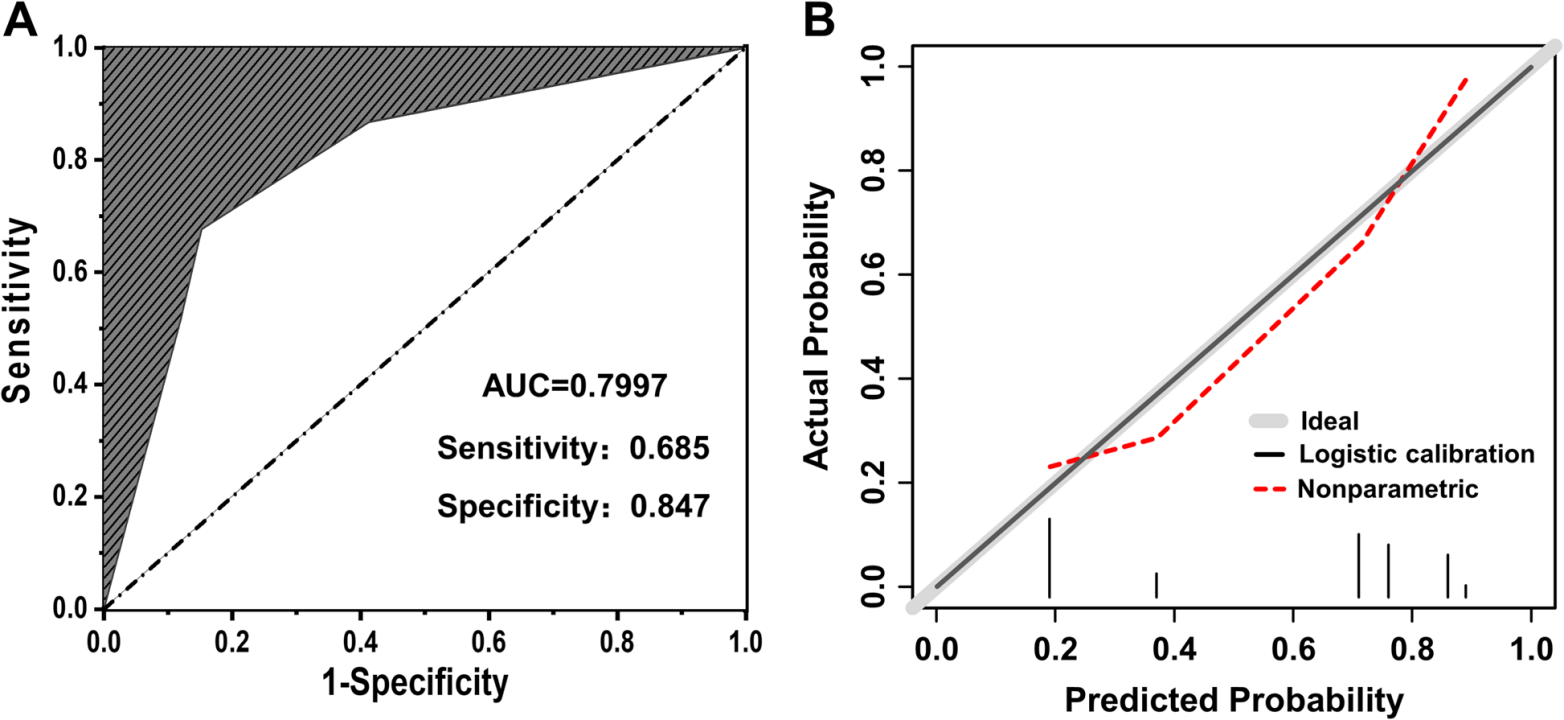
The validation of MVI prediction model. **A** The area under the curve (AUC) for the MVI prediction model. **B** The calibration curve of MVI prediction model

The validation of the MVI prediction model mainly included an assessment of the area under the curve (AUC) and the calibration curve, which are presented in Fig. 4. The AUC of the MVI prediction model was 0.7997 with a sensitivity of 0.685 and specificity of 0.847, indicating good predictive ability. Moreover, the calibration curve of the MVI prediction model presented a good H-L goodness of fit (*P* = 0.231 > 0.05), and showed high coherence between the observed risk and the predicted risk, indicating that the model has a superior predictive performance.

### Overall survival and recurrence comparison between the two groups

**Fig. 5:**
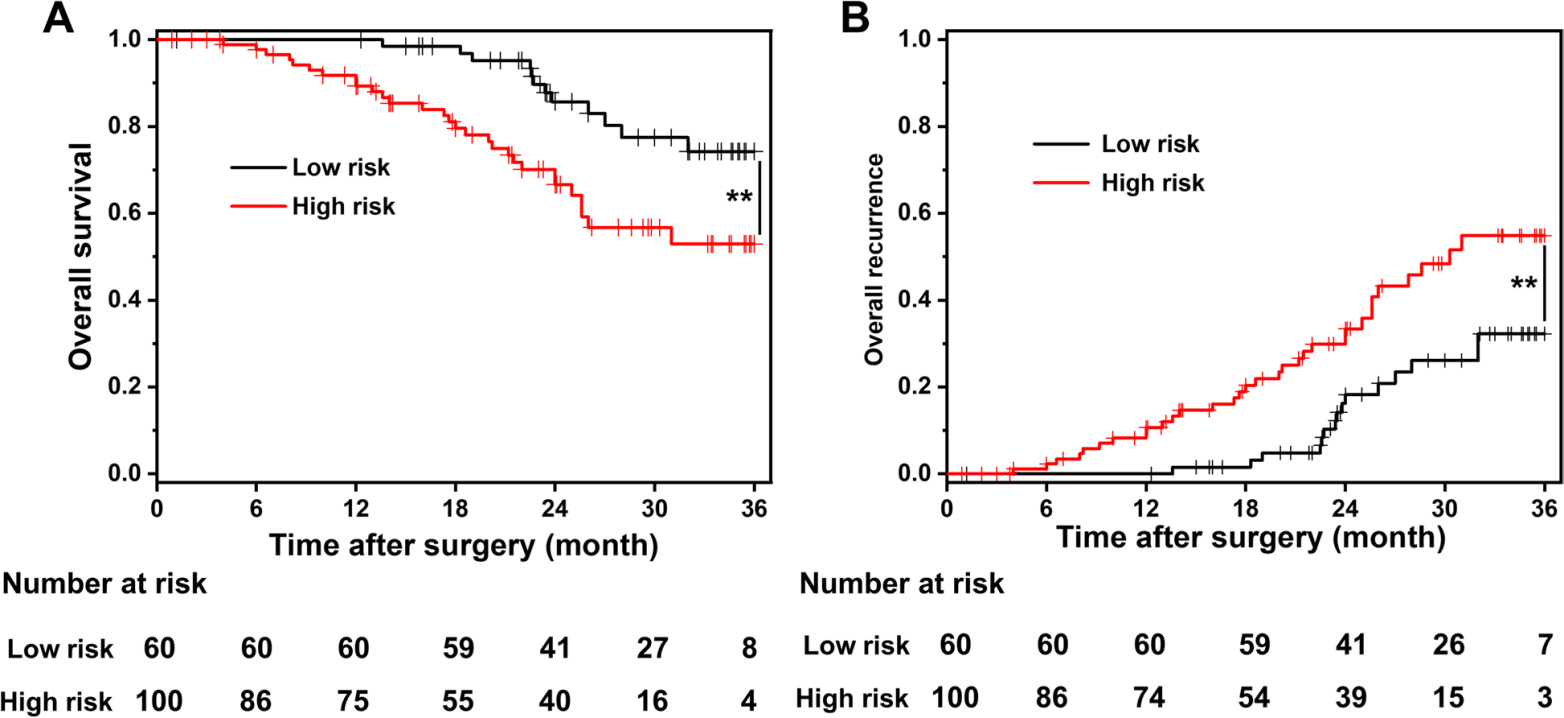
The overall survival and recurrence comparison between the MVI low-risk group and high-risk group. ^**^P < 0.01

According to the cutoff value of 2.5, the patients were classified into the MVI low-risk group (*n* = 60) and the high-risk group (*n* = 100), and the overall survival and recurrence comparison between the two groups is shown in Fig. 5. Compared with the MVI high-risk group, the patients in the MVI low-risk group had a higher survival rate (*P* = 0.002) and a lower recurrence rate (*P* = 0.004), indicating a better clinical prognosis.

## Discussion

Comprehensive treatment based on radical surgery is currently considered the preferred and most effective method for HCC treatment [16, 17]. By eradicating the microscopic metastatic lesions around tumors to a certain extent, the promotion of the concept of anatomic hepatectomy has introduced an ideal surgical means for the radical surgical treatment of HCC [18, 19]. Nevertheless, for MVI-positive patients, even after anatomic hepatectomy, the 5-year recurrence rate is still as high as 60 to 70% [20]. Therefore, preoperative detection of MVI is particularly important. In our study, first, the clinical outcomes and prognosis of clinical HCC patients with or without MVI were compared and analyzed; then, based on preoperative AFP, tumor diameter, and TNM stage, an MVI prediction model with superior predictive efficacy was constructed and validated, which provided a new therapeutic strategy for the comprehensive and standardized treatment of HCC patients.

AFP is an important indicator of HCC screening, which can reflect the occurrence and development of HCC, and is positively correlated with the pathological HCC process [21, 22]. AFP was proven to be an independent risk factor for early recurrence and poor overall survival of HCC patients after hepatectomy, and the association between MVI and AFP has always been a concern for researchers [23]. Patients with AFP ≥ 400 kU/L are usually considered to have a diagnosis of HCC, and AFP ≥ 400 kU/L is also an important risk factor for the early recurrence of HCC [24, 25]. However, a single hematological index is generally not highly specific and its clinical application is limited.

Several studies have confirmed that during the growth process of tumors, poorly differentiated areas gradually replace well-differentiated areas, thus increasing the degree of malignancy and invasiveness of the tumors [26]. Furthermore, a large tumor size stimulates invasive behavior, such as local, lymphatic, and distant metastases [27]. Anatomic hepatectomy has been shown to be significantly beneficial for long-term survival in patients with tumors > 5 cm in diameter, which also suggests that the occurrence of MVI is more likely from tumors with larger diameters [28, 29]. The tumor diameter and TNM stage based on the imaging data are also independent risk factors for postoperative MVI [30, 31]. A larger tumor size might be associated with capsular invasion, satellite nodules, and tumor thrombus, and an advanced TNM stage might be relevant to the degree of aggressiveness and malignancy of the tumor [32, 33]. In our MVI prediction model, preoperative AFP ≥ 400 kU/L, TD ≥ 5 cm, and TNM stage III–IV demonstrated a reasonable predictive ability of MVI.

Currently, radiometric technology is used to extract predictive MVI models from CT images, and hematological indicators have been proposed as predictors [34, 35]. Some studies have found that special features of MRI can be used as typical features of MVI imaging diagnosis, such as cystic insufficiency, coronary enhancement in the arterial phase, and peritumoral low signal [36, 37]. However, the process is quite complex, because radiomic feature extraction requires algorithms developed by scientists and engineers, and many algorithms are difficult to recognize and put into clinical practice due to their overlapping parameters or inadequate imaging. MVI status can also be reflected by specific clinical hematologic indicators including des-gamma carboxy prothrombin (DCP) and peripheral neutrophil to lymphocyte ratio (NLR) [38, 39]. Unfortunately, the predictive accuracy of preoperative hematological indices is poor. As reported in the review [15], many scholars focus on a single indicator, such as tumor size, PIVKA-II level, or pure hematological/radiological indicators, to construct MVI prediction models, but unfortunately, the practicability and feasibility of these models in clinical practice remains to be further verified. In our prediction model, based on the risk factors derived from hematological data, imaging data, and clinical staging assessment, it achieved a high AUC of 0.7997 (with a sensitivity of 0.685 and specificity of 0.847) and good utility (high H-L goodness of fit, *P* = 0.231). By successfully identifying high-risk patients, this prediction model can comprehensively screen tumor occurrence and invasiveness to preoperatively predict MVI in HCC patients.

In addition, our study proved that HCC patients with poor differentiation had a higher incidence of MVI than patients with high/moderate differentiation, which also lead to a poor prognosis, which is consistent with previous studies. Poorly differentiated tumors are more aggressive than well-differentiated tumors, so the prognosis of patients with poorly differentiated HCC after resection is worse than that of patients with highly differentiated HCC, and the possibility of postoperative recurrence is increased [40, 41]. Although MVI positivity showed no significant difference in intraoperative and postoperative clinical indicators, the difference in clinical prognosis can effectively guide further comprehensive treatment strategies. MVI is an independent risk factor for a poor prognosis after radical resection of HCC [42, 43]. Therefore, through accurate prediction of preoperative MVI, more reasonable and timely treatment options can be selected clinically, including radiofrequency ablation, anatomic hepatectomy, or liver transplantation, and even the choice between neoadjuvant and adjuvant therapy can be extended. In addition, although targeted therapy for MVI is the first choice in the traditional sense, targeted therapy should also include a systematic treatment method for MVI to truly achieve personalized treatment for patients based on tumor biological behavior [44–46].

This study also has some limitations. Some studies have found that the margin of the tumor to the surgical margin plane can significantly affect postoperative outcomes, but these studies were not further discussed in the survival analysis. Although the sample size included in this study reached the standard for statistical analysis, it is necessary to carry out a randomized controlled study with a large sample for analysis and research on the complex etiology of HCC recurrence. In addition, the follow-up time of this study was 36 months, so it is necessary to carry out a longer follow-up of patients and analyze the mortality and recurrence rates at different stages to study the relationship between MVI and the prognosis of HCC patients in greater detail.

## Conclusion

We conducted a retrospective study to verify that HCC patients with pathologically poor differentiation had a higher incidence of MVI, which was also related to a poor prognosis after radical surgery. Then, we constructed and validated an MVI prediction model for HCC patients based on preoperative AFP, tumor diameter, and TNM stage, which presented superior predictive efficacy and strong clinical practicability. We concluded that MVI was an independent risk factor for a poor prognosis after radical resection, and HCC patients with a high risk of MVI should receive more reasonable and timely comprehensive treatment due to early recurrence and poor survival after radical surgery. This study provides a new therapeutic strategy for accurate clinical prognostic judgment and standardized treatment of HCC patients.

## Data Availability

Due to privacy and ethical restrictions, the data are not publicly available.
